# Human brown fat metabolism associates with systemic branched-chain amino acids homeostasis

**DOI:** 10.1016/j.molmet.2026.102434

**Published:** 2026-08-26

**Authors:** Mueez U-Din, Francisco M. Acosta, Teemu Saari, Vanessa D. de Mello Laaksonen, Birgitta W. van der Kolk, Milena Monfort-Pires, Mercedes Clemente-Postigo, Tarja Niemi, Markku Taittonen, Marjo Tuomainen, Tobias Fromme, Marko Lehtonen, Karsten Kristiansen, John W. Newman, Kirsi H. Pietiläinen, Jussi Pihlajamäki, Eija Pirinen, Martin Klingenspor, Kati Hanhineva, Juho Raiko, Kirsi A. Virtanen

**Affiliations:** 1 Turku PET Centre, Turku University Hospital, Turku, Finland; 2 Turku PET Centre, University of Turku, Turku, Finland; 3 InFLAMES Research Flagship Centre, University of Turku, Turku, Finland; 4 MediCity Research Laboratories, University of Turku, Turku, Finland; 5 Department of Public Health and Clinical Nutrition, University of Eastern Finland, Kuopio, Finland; 6 Obesity Research Unit, Research Program for Clinical and Molecular Metabolism, Faculty of Medicine, University of Helsinki, Helsinki, Finland; 7 Healthy Weight Hub, Abdominal Center, Helsinki University Hospital and University of Helsinki, Helsinki, Finland; 8 Department of Cell Biology, Genetics and Physiology, Faculty of Science, University of Malaga, Malaga, 29010, Spain; 9 Unidad de Gestión Clínica Endocrinología y Nutrición, Instituto de Investigación Biomédica de Málaga-Plataforma en Nanomedicina (IBIMA-Plataforma Bionand), Hospital Universitario Virgen de la Victoria, Universidad de Málaga, Malaga, 29010, Spain; 10 CIBER Physiopathology of Obesity and Nutrition (CIBEROBN), Carlos III Health Institute, Madrid, 28029, Spain; 11 Department of Plastic and General Surgery, Turku University Hospital, Turku, Finland; 12 Department of Anesthesiology, Turku University Hospital, Turku, Finland; 13 Chair for Molecular Nutritional Medicine, Technical University of Munich, Freising, Germany; 14 EKFZ - Else Kröner Fresenius Center for Nutritional Medicine, Technical University of Munich, Freising, Germany; 15 ZIEL - Institute for Food & Health, Technical University of Munich, Freising, Germany; 16 School of Pharmacy, University of Eastern Finland, Kuopio, Finland; 17 Department of Biology, University of Copenhagen, Copenhagen, Denmark; 18 Obesity and Metabolism Research Unit, USDA-ARS Western Human Nutrition Research Center, Davis, CA, USA; 19 West Coast Metabolomics Center, Davis Genome Center, University of California, Davis, Davis, CA, 95616, USA; 20 Department of Nutrition, University of California, Davis, Davis, CA, 95616, USA; 21 Department of Medicine, Endocrinology and Clinical Nutrition, Kuopio University Hospital, Wellbeing Services County of North Savo, Kuopio, Finland; 22 Research Program for Clinical and Molecular Metabolism, University of Helsinki, Finland; 23 Faculty of Medicine, Research Unit of Biomedicine and Internal Medicine, University of Oulu, Oulu, Finland; 24 Medical Research Center Oulu, Oulu University Hospital and University of Oulu, Oulu, Finland; 25 Biocenter Oulu, University of Oulu, Oulu, Finland; 26 Department of Life Technologies, Food Chemistry and Food Development Unit, University of Turku, Turku, Finland

**Keywords:** Brown adipose tissue, Branched-chain amino acids, Insulin resistance

## Abstract

Circulating branched-chain amino acids (BCAAs) are linked with insulin resistance, but the human tissues contributing to systemic BCAA homeostasis remain incompletely defined. Brown adipose tissue (BAT) is a metabolically active adipose depot associated with favourable insulin sensitivity, yet its role in BCAA metabolism in humans remains unclear. We tested whether human BAT metabolism is associated with circulating BCAA levels, BAT-resident BCAA-catabolic signatures, and longitudinal changes in systemic BCAA homeostasis. We studied 83 adults who underwent metabolic phenotyping, PET-CT assessment of cold-stimulated BAT metabolism, and serum metabolomic profiling at room temperature and during acute mild cold exposure. Supraclavicular BAT biopsies from 25 participants were analysed by transcriptomics and metabolomics, and 40 participants were re-examined for circulating BCAA profiles after approximately five years. Participants with high BAT metabolism had lower circulating BCAA levels than those with low BAT metabolism. Within BAT, metabolically active individuals exhibited lower relative BCAA abundance together with higher expression of genes involved in BCAA catabolism. These BAT BCAA-catabolic signatures aligned with thermogenic capacity and indices of systemic insulin sensitivity. In contrast, individuals with low BAT metabolism showed increases in circulating BCAAs over five years. Integrative analyses further linked circulating lipopolysaccharide, a marker of metabolic endotoxemia, with higher BAT BCAA and aminomalonate abundance, together with transcriptional patterns involving inflammatory and mitochondrial pathways. Together, these findings identify human BAT metabolism as a tissue phenotype linked to systemic BCAA homeostasis and extend the role of human BAT beyond thermogenesis, suggesting that BAT-associated BCAA handling may contribute to systemic metabolic health.

## Highlights

• Humans with highly active BAT exhibit lower circulating and BAT-resident BCAAs.
• BAT BCAA-catabolic signatures align with systemic insulin sensitivity.
• Low BAT metabolism is linked to rising circulating BCAAs over five years.
• Metabolic endotoxemia associate with higher BCAA levels and aminomalonate accumulation in human BAT.

## Graphical abstract

**Figure:**
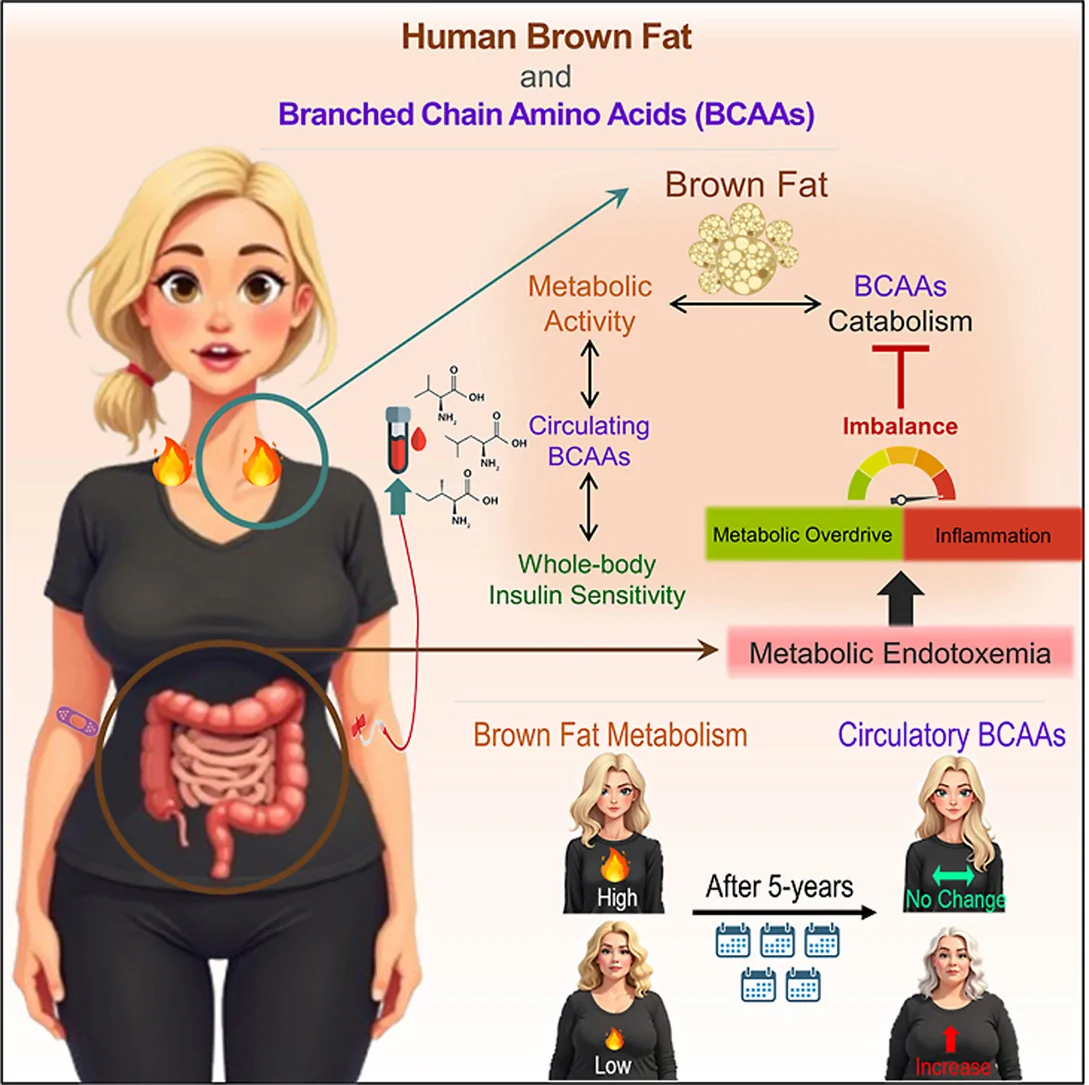

## 1 Introduction

Increasing evidence indicates that active brown adipose tissue (BAT) in humans is associated with a favourable metabolic profile, including better whole-body insulin sensitivity and reduced risk of cardiovascular disease, independent of adiposity [[1], [2], [3]]. BAT has traditionally been recognised as a thermogenic tissue that supports non-shivering thermogenesis during cold exposure and after meals [4]; however, despite its high oxidative metabolism per unit mass, its relatively small mass in adult humans has left its net contribution to systemic energy metabolism uncertain [4,5]. Beyond thermogenesis, BAT is increasingly recognised as a metabolic hub that participates in systemic regulation of branched-chain amino acids (BCAAs: leucine, isoleucine, valine) through uptake and oxidation, thereby impacting whole-body metabolism [6].

Clinically, elevated circulating BCAAs have long been associated with obesity and insulin resistance [7,8], and rodent studies have demonstrated that adipose tissue is a key regulator of systemic BCAA levels [9]. BAT has emerged as a critical site for BCAA metabolism: in both mice and humans, BAT actively oxidizes BCAAs during cold exposure, promoting systemic BCAA clearance [6]. Mechanistic studies in mice show that impaired mitochondrial BCAA catabolism in BAT causes systemic insulin resistance [10], while favourable gut microbiota can supress inflammation in BAT leading to improved BAT BCAA catabolism [11]. Although these studies highlight key mechanisms, the extent to which they operate in humans remains unclear.

Emerging human evidence points to supraclavicular BAT as a metabolically active site for BCAA utilization: elderly males with prostate adenocarcinoma demonstrate higher net uptake of ^18^F-fluciclovine (a leucine analogue PET tracer) in supraclavicular BAT than in abdominal subcutaneous fat, and individuals with obesity exhibit higher expression of genes mediating mitochondrial BCAA uptake and catabolism in this depot [12]. Despite several limitations in these human studies, these findings indicate that BAT may utilise BCAA more than white adipose tissue (WAT) and may contribute to systemic BCAA homeostasis.

Although previous human studies have contrasted BAT with WAT [6,12], a major knowledge gap concerns heterogeneity within BAT itself. Specifically, it is unknown whether individuals with high BAT metabolism differ from those with low BAT metabolism in: (i) circulating BCAA profiles; (ii) BAT-resident BCAA content; or (iii) expression of genes regulating BCAA catabolism. Beyond cross-sectional differences, the longitudinal relevance of BAT metabolism for maintaining systemic BCAA homeostasis and metabolic health remains unexplored. Furthermore, it is unclear which BAT-specific metabolic steps, including TCA cycle flux, constrain local or systemic BCAA accumulation, or whether inflammatory factors such as metabolic endotoxemia influence BAT BCAA catabolism. Addressing these questions in healthy humans of both sexes is critical for defining how variation in BAT metabolism contributes to BCAA homeostasis and systemic metabolic regulation.

Here, by integrating PET imaging, multi-omics profiling of human BAT, and extensive metabolic phenotyping with longitudinal follow-up, we directly test how inter-individual variation in BAT metabolism shapes local and systemic BCAA metabolism and its impact on metabolic health.

## 2 Methods

### 2.1 Study participants

A total of 83 participants were included in this study. Data and biological samples were collected between 2009 and 2018 at the Turku PET Centre as part of clinical studies investigating human BAT metabolism [3,5,13,14]. Participants were men and women aged 20–55 years with BMI ranging from 19 to 44 kg/m^2^. All underwent clinical evaluation prior to enrolment, and only individuals with a metabolically healthy profile, without diabetes or cardiovascular disease, were included. Health status was determined using medical records, a 2-h oral glucose tolerance test, electrocardiography, and clinical lipid and hepatic enzyme measurements. All participants provided written informed consent. Study protocols were approved by the Ethics Committee of the Hospital District of Southwest Finland and conducted in accordance with the Declaration of Helsinki.

### 2.2 High- and Low-BAT classification

Participants were categorized as having high or low BAT metabolism based on cold-stimulated substrate uptake measured by PET imaging, using glucose or non-esterified fatty acid (NEFA) uptake as indicators of cold-induced BAT activation. Individuals with a cold-stimulated glucose uptake rate ≥3.0 μmol/100 g/min or NEFA uptake rate ≥0.7 μmol/100 g/min were classified as high-BAT, whereas those below these thresholds were assigned to the low-BAT group [3].

### 2.3 BAT biopsies and PET imaging

Supraclavicular BAT biopsies were obtained after written informed consent. The BAT depot was localized using available imaging data, including MRI or cold-exposed [^18^F]FDG or [^18^F]FTHA PET scans. From the same incision site, subcutaneous adipose tissue was collected as a WAT reference. Biopsies were performed at room temperature (∼22 °C) under local lidocaine–epinephrine anaesthesia by a plastic surgeon. Immediately after excision, tissue samples were snap-frozen in liquid nitrogen and stored at −80 °C until analysis.

### 2.4 RNA isolation, library preparation, and sequencing

Deep-frozen adipose tissue samples (30–120 mg) were homogenized in 1 mL TRIsure reagent (Bioline, UK) using an Ultra-Turrax disperser (Miccra GmbH, Germany). Total RNA was isolated and purified using spin columns (SV Total RNA Isolation System, Promega, USA) and eluted in 50 μL of nuclease-free water. RNA concentration and purity were determined spectrophotometrically (Nanodrop ND-1000, Peqlab, Germany), and integrity was verified using a Bioanalyzer 2100 (Agilent Technologies, USA). Coding RNA libraries were prepared from 25 to 500 ng RNA using the TruSeq RNA Access Library Prep Kit (Illumina, USA) and sequenced on a HiSeq 2500 platform (Illumina) as previously described [4]. Gene expression levels were quantified as reads per kilobase of transcript per million mapped reads (RPKM). Genes of interest were selected based on the KEGG pathways for BCAA, Glutathione, Toll-like receptor signalling and NF-kappa B signalling [[15], [16], [17], [18]] for hypothesis-driven statistical analyses.

### 2.5 BCAA, TCA cycle and ETC pathway scoring

Gene expression values for enzymes involved in BCAA catabolism, the tricarboxylic acid (TCA) cycle, and the electron transport chain (ETC) were standardized across participants by z-score transformation, defined as,

$$z=\frac{x-\mu }{\sigma }$$

where x is the raw expression value, μ the mean, and σ the standard deviation across participants.

For the TCA cycle, overall pathway scores were computed as the mean z-score across all genes, and step-specific scores as the mean z-score of genes representing each enzymatic step (pyruvate dehydrogenase, citrate synthase, aconitase, isocitrate dehydrogenase, α-ketoglutarate dehydrogenase, succinyl-CoA synthetase, succinate dehydrogenase, fumarase, and malate dehydrogenase).

ETC genes were grouped by complex: Complex I (*NDUF* family), Complex II (*SDHB*, *SDHC*, *SDHD*), Complex III (*UQCR*, *CYCS*), and Complex IV (*COX* family and assembly factors). Complex-specific scores were calculated as the mean z-score within each complex, and an overall ETC score was obtained by averaging across all genes, including *CYB5A*.

For BCAA catabolism, genes were categorized into transporters (*SLC3A2*, *SLC7A5*, *SLC7A8*), transaminases (*BCAT1*, *BCAT2*), the BCKDH complex and its regulators (*BCKDHA*, *BCKDHB*, *DBT*, *DLD*, *BCKDK, PPM1K*), valine catabolism (*ACAD8, HIBCH, ALDH6A1*), and leucine/isoleucine catabolism (*IVD, MCCC1, MCCC2, HMGCL*). Category-specific scores were defined as the mean z-score within each functional group, and an overall BCAA catabolism score was computed as the average z-score across all genes.

### 2.6 Serum metabolomics

Fasting blood samples obtained during acute cold exposure were analysed for circulating serum BCAAs as previously described [19]. Briefly, samples were deproteinised with acetonitrile, and analysed using liquid chromatography high-resolution mass spectrometry (UHPLC–qTOF-MS). Untargeted metabolomics analysis was performed using two complementary chromatographic techniques, namely reversed-phase chromatography and hydrophilic interaction chromatography. Furthermore, the samples were analysed under both positive and negative electrospray ionization modes.

For fasting blood samples collected at room temperature, including samples taken at 5-years follow-up, circulating BCAAs were quantified in serum using the Nightingale Health ^1^H NMR metabolomics platform (Nightingale Health Ltd, Helsinki, Finland), which provides high-throughput absolute quantification of amino acids with established analytical reproducibility.

### 2.7 Tissue metabolomics

BAT samples were extracted in 1 mL of ice-cold acetonitrile:isopropanol:water (3:3:2, v/v/v). Extracts were vortexed and centrifuged, and half of the supernatant was dried to completeness under vacuum prior to derivatization.

Dried extracts were first methoximated in 10 μL of methoxyamine hydrochloride (40 mg/mL in pyridine) at 30 °C for 90 min with continuous agitation. Samples were subsequently silylated by adding 91 μL of N-methyl-N-(trimethylsilyl)trifluoroacetamide (MSTFA) containing fatty acid methyl ester (FAME) retention index standards, followed by incubation at 37 °C for 30 min. Derivatised samples were transferred to autosampler vials and sealed prior to analysis.

Metabolomics profiling was performed using an Agilent 7890A gas chromatograph coupled to a LECO time-of-flight mass spectrometer (GC–TOF-MS). A 0.5 μL aliquot of derivatised sample was injected in splitless mode onto a RESTEK RTX-5SIL MS column equipped with an Integra-Guard pre-column. The injector temperature was set to 275 °C with helium as the carrier gas at a constant flow of 1 mL/min. The GC oven was held at 50 °C for 1 min, ramped at 20 °C/min to 330 °C, and maintained at 330 °C for 5 min. The transfer line temperature was set to 280 °C, and the electron impact ion source was maintained at 250 °C.

Mass spectra were acquired in electron impact mode over a mass-to-charge (*m*/*z*) range of 85–500 at an acquisition rate of 17 spectra per second. Metabolites annotated by mass spectrometry (citric acid, succinic acid, fumaric acid, malic acid) are reported in text and figures as their physiological forms (citrate, succinate, fumarate, malate).

### 2.8 Circulating LPS measurement (metabolic endotoxemia)

Circulatory LPS was measured on two separate day and conditions, 1 day at RT and once during cold stimulation. The samples in both of these days were acquired after an overnight fast. An average of these two days was considered as a marker for metabolic endotoxemia. For LPS quantification, serum samples were thawed and measured using the limulus amebocyte lysate (LAL) chromogenic endpoint assay (Hycult Biotech, Uden, The Netherlands) according to the manufacturer’s protocol. Samples were diluted in pyrogen-free water and heated at 70 °C for 10 min to inactivate agents that could neutralize endotoxin activity in the LAL assay. To assess potential assay interference, samples were spiked, testing for inhibition or enhancement. All measurements were performed in duplicate, and results were considered valid when the intra-assay coefficient of variation was within ±10%. Extreme precautions were taken to prevent endotoxin contamination, and all materials used for sample handling and analysis were pyrogen-free.

### 2.9 Hepatic insulin resistance index (HIRI)

HIRI was calculated from the early phase of the OGTT, which predominantly reflects hepatic insulin action. Plasma glucose and insulin concentrations at fasting (0 min), 15, and 30 min were used to compute area under the curve (AUC) using the trapezoidal method. The index was defined as:

$$HIRI=Glucose{AUC}_{0-30}\times Insulin{AUC}_{0-30}$$

### 2.10 Muscle insulin resistance index (MIRI)

MIRI was derived from the late phase of the OGTT, representing predominantly peripheral (skeletal muscle) insulin action. Plasma glucose and insulin concentrations at 60, 90, and 120 min were used to calculate AUCs using the trapezoidal method. MIRI was defined as:

$$MIRI=Glucose{AUC}_{60-120}\times Insulin{AUC}_{60-120}$$

### 2.11 Adipose tissue insulin resistance (Adipo-IR)

Adipose tissue insulin resistance was estimated using fasting plasma values. Adipo-IR reflects the ability of insulin to suppress lipolysis in adipose tissue. It was calculated as:

AdipoIR=FastingFFA×FastingInsulin

### 2.12 Data analysis

Comparisons between High-BAT and Low-BAT groups under cold or room-temperature (RT) conditions were performed using unpaired two-tailed Student’s *t*-tests for normally distributed data, or Mann–Whitney *U* tests when normality was not met. Correlations between variables were assessed using Spearman’s rank correlation. A *p*-value <0.05 was considered statistically significant. Data are presented as mean ± SD unless otherwise indicated, and *N* denotes the number of participants. All data processing and statistical analyses were conducted using R (version 4.3.0 or later) and IBM SPSS Statistics, with data visualization performed in GraphPad Prism, SPSS, and R.

## 3 Results

The study included 83 healthy study volunteers of both genders with mean body mass index (BMI) of 26.8 ± 5.5 kg m^−2^ (Range: 19–44 kg m^−2^) and mean age of 39 ± 10 years (range: 20–55 years). Among these study participants, 25 provided biopsies of supraclavicular BAT for the biochemical analysis. The study participants were classified either to have High-BAT or Low-BAT metabolism based on the extent of cold-stimulated substrate uptake rates measured with PET imaging [3]. The details of study participants have been provided in the supplemental information (Supplemental Tables S1–S3).

### 3.1 Circulating BCAAs associate with BMI and insulin sensitivity, and differ by Brown fat metabolism

**Figure 1:**
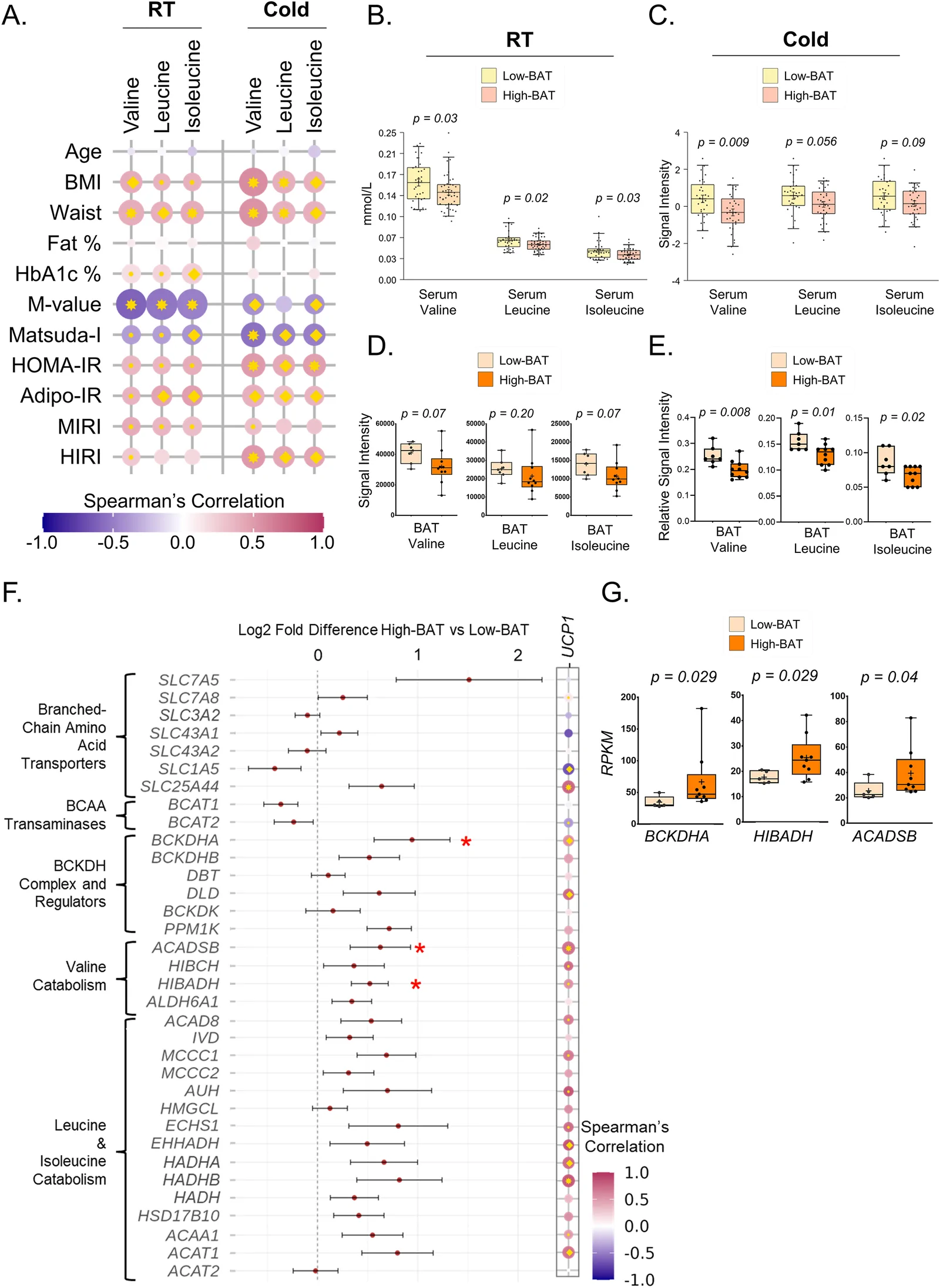
C**irculating and BAT-resident BCAAs and BCAA-pathway gene expression in humans with High-BAT and Low-BAT metabolism**. **A.** Correlations between serum branched-chain amino acid (BCAA) concentrations measured at room temperature and during cold exposure, and anthropometric indices, body adiposity, and markers of whole-body insulin sensitivity and resistance (Adipo-IR: adipose tissue insulin resistance index; MIRI: muscle insulin resistance index; HIRI: hepatic insulin resistance index). Significant associations are highlighted with a yellow marker (• *p* < 0.05, ◆ *p* < 0.01, ✸ *p* < 0.001). **B–C.** Comparison of serum BCAA levels at room temperature (**B**) and during cold exposure (**C**) between individuals with High-BAT and Low-BAT metabolism. Box plots show the median (centre line) and interquartile range (box); whiskers extend to 1.5x the interquartile range. Individual data points are shown. **D–E.** BAT-resident BCAAs (**D**) and their relative abundances (**E**) in High-BAT versus Low-BAT groups. Box plots show median and interquartile range; whiskers indicate minimum–maximum values. Individual data points are shown. **F**. Log_2_ fold differences in the expression of genes involved in BCAA transport, transamination, BCKDH complex activity, and downstream catabolic pathways, and their relationship with BAT *UCP1* expression. Dots represent log_2_ fold difference between High-BAT and Low-BAT groups, and error bars indicate SEM. Genes with significant differences are denoted with ∗ (*p* < 0.05). Significant correlations are indicated with a yellow marker (• *p* < 0.05, ◆ *p* < 0.01, ✸ *p* < 0.001). **G**. Expression of *BCKDHA*, *HIBADH*, and *ACADSB* in BAT between High-BAT and Low-BAT groups. Box plots show median and interquartile range; whiskers indicate minimum–maximum values. Individual data points are shown.

Serum BCAAs (valine, isoleucine, and leucine) levels, measured from samples collected both at room temperature and under acute mild cold exposure, associated positively with BMI and waist circumference but showed no relationship with age or body fat percentage (Figure 1A). Because percentage body fat represents a ratio-based adiposity index that may incompletely account for body-size effects [20], we additionally examined associations using absolute fat mass with regression-based adjustment for body weight, age, and sex. Total circulating BCAAs were positively associated with adjusted fat mass during cold exposure (*rho = 0.36, p = 0.005*), with a similar but non-significant trend at room temperature (*rho = 0.22, p = 0.06*). Consistently, in multivariable regression models including body weight, age, and sex, fat mass remained an independent predictor of circulating BCAA levels (RT: *p = 0.05*, Cold: *p = 0.01*).

Circulating BCAA levels were inversely associated with whole-body insulin sensitivity as assessed by the hyperinsulinemic–euglycemic clamp (M-value), and positively with HOMA-IR, adipose tissue insulin resistance index (Adipo-IR), hepatic insulin resistance index (HIRI), and muscle insulin resistance index (MIRI, Figure 1A). These findings reinforce prior observations that elevated circulating BCAAs accompany systemic insulin resistance in humans.

Importantly, circulatory BCAAs (both RT concentrations and cold-stimulated signal intensities) were lower in individuals with high BAT metabolism compared with those with low BAT metabolism (Figure 1B–C). However, these differences were attenuated and no longer significant after adjustment for BMI in ANCOVA models (Valine: *p = 0.13*; Leucine: *p = 0.89*; Isoleucine: *p = 0.27*).

Although prior work suggested that acute cold stimulation lowers circulating BCAA levels [6], we could not directly assess dynamic cold responses because RT and cold samples were analysed on separate metabolomics platforms. Nevertheless, RT and cold-stimulated BCAA levels were positively correlated (valine: *rho = 0.52, p* < *0.001*; leucine: *rho = 0.50, p* < *0.001*; isoleucine: *rho = 0.62, p* < *0.001*).

To assess whether the lower circulating BCAA levels in High-BAT individuals reflected a broader difference in serum amino acids, we analysed the available amino acids other than BCAAs. At room temperature, alanine, glutamine, glycine, histidine, and phenylalanine did not differ between High-BAT and Low-BAT groups (Supplementary Figure S1). Coverage of other amino acids in the acute cold-exposure metabolomics was limited; however, serum glutamate also did not differ between groups (Supplementary Figure S1). These findings suggest that the circulating BCAA differences were not explained by a generalized reduction in measured serum amino acids.

Together, these results confirm that circulating BCAA levels increase with adiposity and insulin resistance. Although BCAA levels were lower in individuals with High-BAT metabolism, the between-group differences were attenuated after adjustment for BMI.

### 3.2 BAT-resident BCAA abundance is lower in individuals with High-BAT metabolism

Given that circulating BCAAs reflect a composite of dietary intake, hepatic and adipose metabolism, muscle protein turnover, and renal clearance [9,21,22], we directly assessed BCAA metabolism within human brown fat by performing metabolomics profiling of supraclavicular BAT biopsies. Tissue (BAT) BCAA levels, in contrast to circulatory levels, may reflect local uptake, and intracellular BCAA handling. Individuals with high-BAT metabolism exhibited a trend to lower absolute levels of BCAAs in BAT compared with those with low-BAT metabolism (Figure 1D). Because amino acids compete for cellular uptake, we further examined the relative abundance of BCAAs (BCAAs as a fraction of total amino acids) within BAT depot. Relative BCAA levels were significantly lower in participants with high BAT metabolism (Figure 1E).

We next examined whether the lower relative BCAA abundance reflected a broader shift in the BAT amino-acid pool. Most measured BAT-resident amino acids, other than BCAAs, did not differ between High-BAT and Low-BAT groups except tryptophan (Supplementary Figure S2), while BAT-resident glutamine and lysine showed a trend towards lower abundance (Supplementary Figure S2). Thus, the observation of lower BAT-resident BCAA in High-BAT was not accompanied by a uniform reduction across all measured amino acids.

To determine whether the BAT-resident BCAA phenotype was specific to BAT or reflected a broader adipose tissue amino-acid pattern, we performed parallel analyses in adjacent WAT samples. In contrast to BAT, neither the individual signal intensities nor the relative signal intensities of WAT-resident valine, leucine, and isoleucine differed significantly between High-BAT and Low-BAT individuals (Supplementary Figure S3). Similarly, the broader WAT amino-acid profile did not show a consistent difference between the groups (Supplementary Figure S4).

Together, these findings identify a BAT-enriched pattern of lower tissue BCAA abundance in individuals with High-BAT metabolism, consistent with greater local BCAA utilization or turnover.

### 3.3 BAT BCAA catabolic signature aligns with thermogenic capacity and systemic insulin sensitivity

To elucidate mechanisms underlying the lower BCAA abundance observed in individuals with High-BAT metabolism, we analysed BAT transcriptome stratified by BAT metabolism (High vs Low). Expression of genes encoding BCAA transporter (cellular uptake) did not differ significantly between groups (Figure 1F). In contrast, individuals with high BAT metabolism exhibited higher expression of multiple BCAA catabolic genes, including *BCKDHA*, *HIBADH*, and *ACADSB* (Figure 1F–G). Moreover, expression of mitochondrial BCAA transport (*SLC25A44*) and oxidative enzymes (*BCKDHA*, *DLD*, *ACAD8*, *HADHA*, *HADHB*, *HIBCH*, *HIBADH*, *ACADSB*, *ACAT1*) showed strong positive associations with *UCP1* expression. Together, these findings suggest that the lower BCAA abundance in metabolically active BAT is more consistent with a stronger mitochondrial BCAA-catabolic gene expression profile rather than with differences in cellular BCAA transporters expression.

To determine whether a similar transcriptional pattern was present in adjacent adipose tissue, we examined WAT expression of genes involved in BCAA transport and catabolism (Supplementary Figure S5). Although some individual WAT genes differed or showed trend-level differences between High-BAT and Low-BAT groups, these transcriptional differences were not accompanied by significant reductions in WAT-resident BCAA abundance (Supplementary Figure S3), in contrast to the lower BCAA abundance and higher BCAA-catabolic gene expression observed in BAT (Figure 1D–F). Therefore, these findings suggest that the BCAA-related tissue phenotype is more pronounced in supraclavicular BAT.

We next asked whether the expression of genes involved in BCAA catabolism in BAT relates to systemic metabolic health. The mitochondrial transaminase *BCAT2* expression in BAT was inversely associated with both muscle and hepatic insulin resistance, whereas *SLC43A1* expression correlated positively with insulin sensitivity (M-value, Matsuda index) and inversely with adipose (Adipo-IR), muscle (MIRI), and hepatic (HIRI) insulin resistance indices. *BCKDHA*, a key component of the regulator of irreversible BCAA oxidation, expression showed a strong positive trend with the M-value (*rho = 0.57, p = 0.051*) and an inverse association with Adipo-IR (*rho = −0.59, p = 0.035*). In addition, *BCKDK,* the inhibitory kinase of BCAA oxidation, expression was inversely related to MIRI, and *IVD*, a downstream enzyme in BCAA oxidation, expression was inversely related to HIRI (Supplemental Figure S6).

We additionally examined BAT-resident amino acids previously shown to incorporate BCAA-derived nitrogen: glutamate, alanine, proline, and glycine [10], in relation to insulin sensitivity. BAT alanine correlated positively with the Matsuda index (*rho = 0.52, p = 0.046*) and inversely with MIRI (*rho = −0.54, p = 0.040*) and Adipo-IR (*rho = −0.51, p = 0.038*), whereas the other amino acids showed no significant associations (Supplementary Figure S7).

Together, these findings show that a stronger BCAA-catabolic transcriptional phenotype in human BAT is associated with thermogenic capacity and more favourable systemic insulin sensitivity, supporting a potential contribution of BAT BCAA metabolism to whole-body metabolic health.

### 3.4 BAT-resident BCAA abundance associates with the succinate:fumarate ratio and aminomalonate

**Figure 2:**
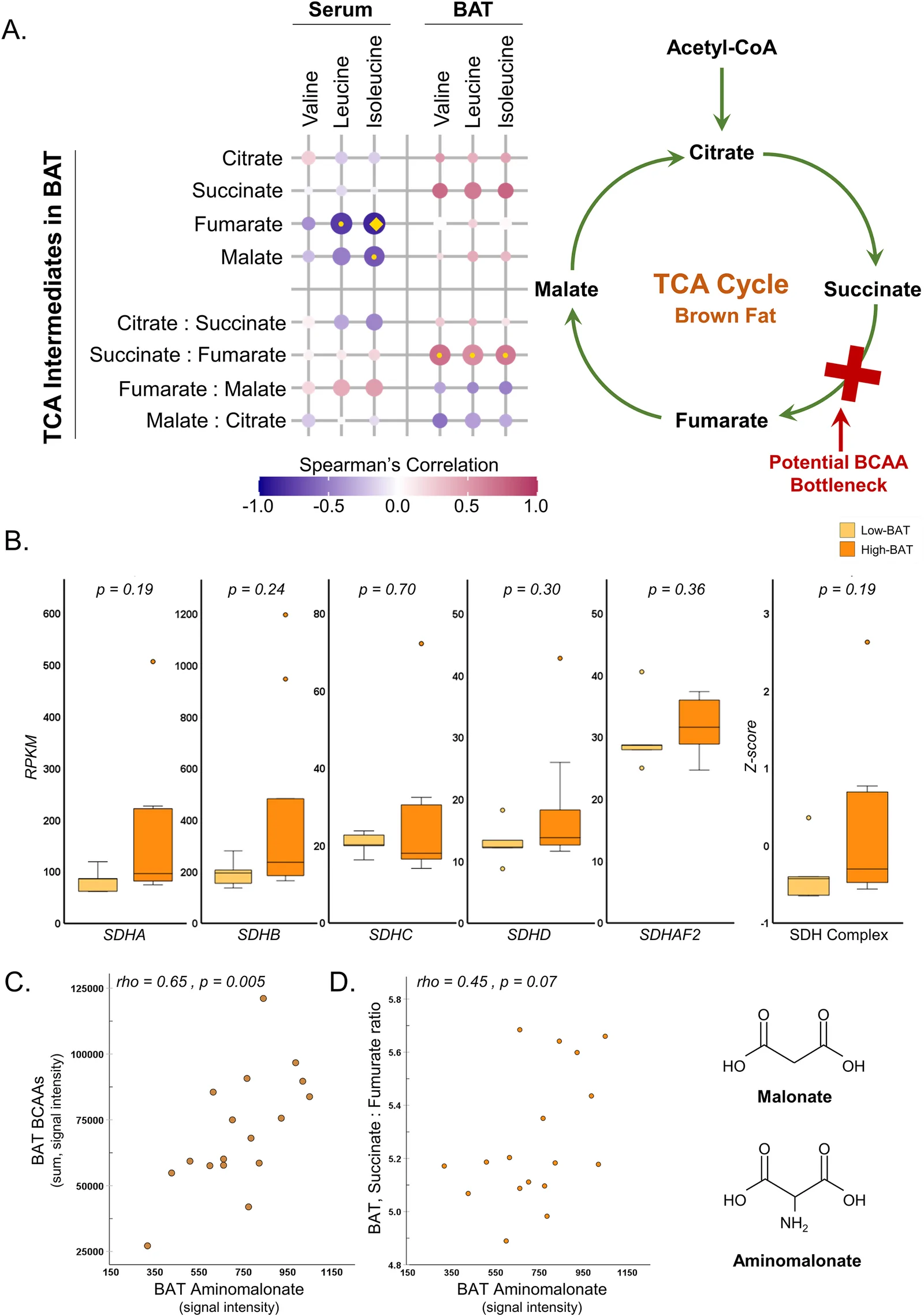
**Serum and BAT-resident BCAAs and their relationship with TCA cycle metabolites in humans with High-BAT and Low-BAT metabolism. A.** Correlations between serum and BAT-resident branched-chain amino acids (BCAAs) and tricarboxylic acid (TCA) cycle intermediates in BAT. Significant associations are highlighted with a yellow marker (• *p* < 0.05, ◆ *p* < 0.01). **B.** Comparison of the expression of *SDHA*, *SDHB*, *SDHC*, *SDHD*, and *SDHAF2* genes, and the composite z-score of the succinate dehydrogenase (SDH) complex in BAT between High-BAT and Low-BAT individuals. Box plots show the median (center line) and interquartile range (box); whiskers extend to 1.5x the interquartile range. Points beyond the whiskers indicate outliers. **C.** Relationship between BAT-resident BCAAs and aminomalonate signal intensity in BAT. **D.** Relationship between the BAT succinate-to-fumarate ratio and aminomalonate signal intensity in BAT.

Having identified lower BAT-resident BCAA abundance together with a higher expression of BCAA-catabolic genes in individuals with High-BAT metabolism, we next asked how BCAA abundance relates to mitochondrial carbon metabolism within BAT. We therefore examined associations of circulating and BAT-resident BCAAs with measured tricarboxylic acid (TCA)-cycle intermediates and metabolite ratios in BAT. Circulating leucine and isoleucine concentrations were inversely correlated with BAT fumarate and malate levels (leucine vs. fumarate: *rho = −0.72, p = 0.008*; isoleucine vs. fumarate: *rho = −0.73, p = 0.007*; isoleucine vs. malate: *rho = −0.66, p = 0.018)*. Within BAT, valine, leucine, and isoleucine abundances were positively associated with the succinate:fumarate ratio (Figure 2A). This consistent relationship raised the possibility that higher BAT BCAA abundance may be accompanied by a relative constraint at the succinate-to-fumarate step, directing our attention to SDH as a potential link between BCAA abundance and mitochondrial metabolism. However, because these measurements represent steady-state metabolite levels, the succinate:fumarate ratio cannot establish altered SDH activity or TCA-cycle flux.

To examine whether this potential constraint at the succinate-to-fumarate step was accompanied by differences in SDH-complex expression, we compared transcript levels of *SDHA*, *SDHB*, *SDHC*, and *SDHD* between individuals with High-BAT and Low-BAT metabolism. Expression of *SDH* subunits did not differ significantly between the groups (Figure 2B), suggesting that the association between BAT BCAA abundance and the succinate:fumarate ratio was not explained by differences in transcript abundance of the measured SDH-complex components.

Because malonate, a structural analogue of succinate, is a well-established competitive inhibitor of *SDH* activity [23,24], we next inspected for malonate or malonate-like metabolites in BAT. Malonate was not detected in BAT, whereas aminomalonate was detected. Aminomalonate is an amino-substituted analogue of malonate and thus retains a related dicarboxylate structure. This structural relationship prompted us to examine whether aminomalonate associates with the observed BAT-resident BCAA pattern, although structural similarity alone does not establish an effect on SDH activity. BAT aminomalonate abundance was positively correlated with the sum of BAT-resident BCAAs (Figure 2C) and showed a trend toward positive association with succinate:fumarate ratio (Figure 2D).

Together, these findings indicate that higher BAT BCAA abundance coincides with greater aminomalonate abundance and a higher succinate:fumarate ratio, while the origin and functional significance of aminomalonate remain unresolved.

### 3.5 Metabolic endotoxemia associates with BAT-resident BCAA abundance

Previous rodent work demonstrated that the gut microbes *Bacteroides vulgatus* and *Bacteroides dorei* enhance BCAA catabolism in BAT [11], while human studies have linked these taxa to reduced intestinal lipopolysaccharide (LPS) production [25]. Building on these findings, we asked whether low-grade metabolic endotoxemia, reflected by higher circulating LPS concentrations, relates to altered BCAA metabolism in human BAT, potentially revealing a gut–BAT axis.

**Figure 3:**
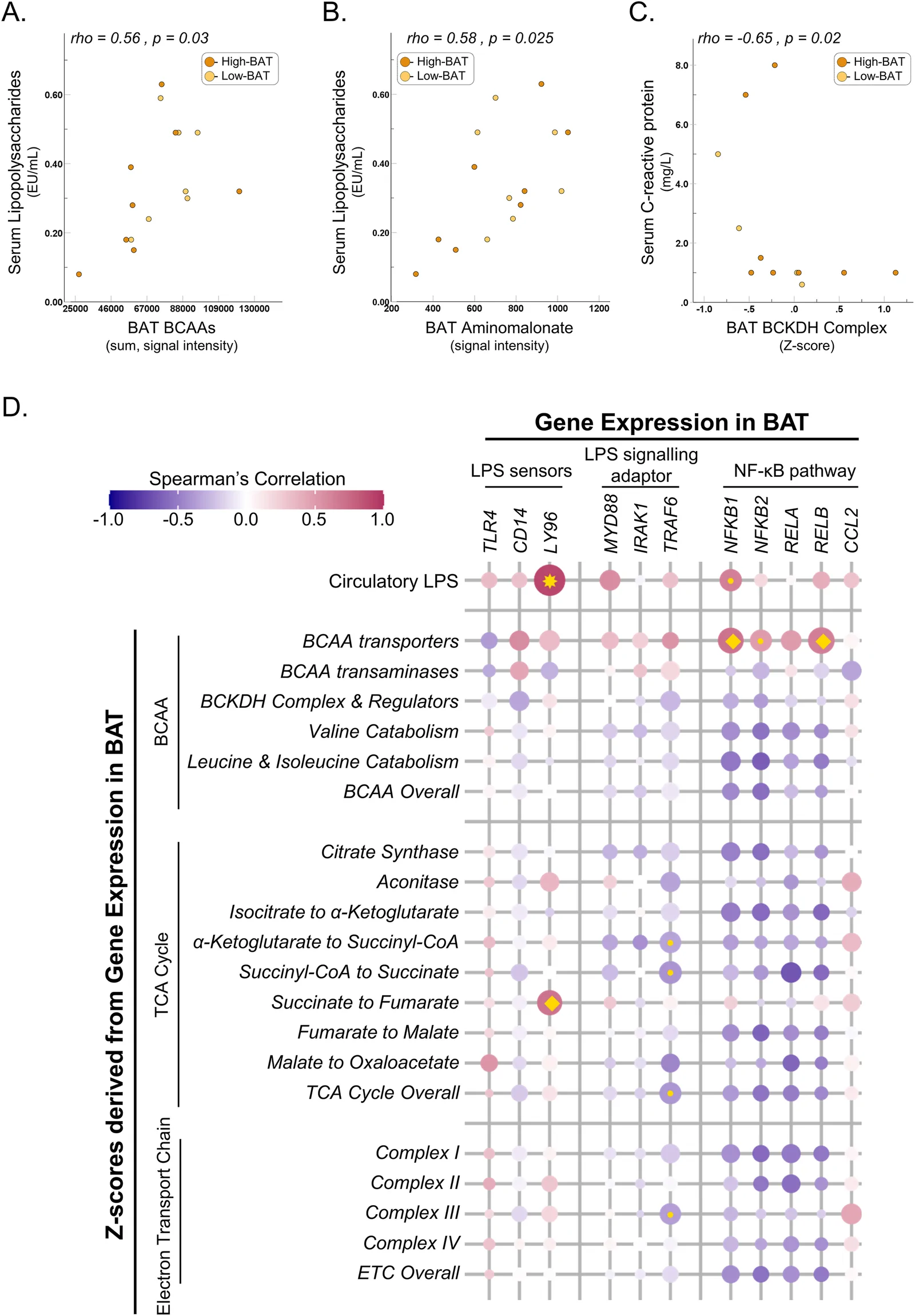
**Circulatory endotoxemia and inflammatory markers in relation to BAT BCAA metabolism and mitochondrial pathways. A.** Correlation between metabolic endotoxemia (circulatory lipopolysaccharides) and BAT-resident branched-chain amino acids (BCAAs). **B.** Correlation between metabolic endotoxemia (circulatory lipopolysaccharides) and aminomalonate signal intensity in BAT. **C.** Association between serum C-reactive protein (CRP) levels and the composite z-score of the BCKDH complex in BAT. **D.** Relationships between the expression of genes related to lipopolysaccharide metabolism (LPS sensors, signalling adaptors, and NF-κB pathway) and the composite z-scores of genes involved in BCAA uptake and catabolism, the tricarboxylic acid (TCA) cycle, and the electron transport chain in BAT. Significant associations are highlighted with a yellow marker (• *p* < 0.05, ◆ *p* < 0.01, ✸ *p* < 0.001).

Circulating LPS concentrations correlated positively with the sum of BCAA in BAT (Figure 3A; individual BCAA, valine*: rho = 0.54, p = 0.039*; leucine: *rho 0.74, p = 0.002*; *isoleucine: rho = 0.52, p = 0.05*). In contrast, circulating LPS was not associated with BCAA abundance in in WAT (Supplementary Figure S8). This BAT-enriched relationship suggests that its high oxidative and mitochondrial capacity may render BAT particularly sensitive to LPS-associated metabolic perturbations. Notably, no correlation was found between circulating LPS and circulating BCAA concentrations, suggesting that the observed relationship is more evident at the tissue level than in the circulation.

In parallel, circulating LPS levels correlated directly with BAT aminomalonate abundance (Figure 3B), suggesting that greater metabolic endotoxemia is associated with higher aminomalonate abundance in BAT.

Supporting a broader inflammatory influence on BCAA metabolism, serum C-reactive protein (CRP), a marker of systemic inflammation, was inversely associated with the composite z-score of genes encoding the BCKDH complex, the rate-limiting step in BCAA catabolism (Figure 3C).

To explore potential mechanisms, we first examined whether circulating LPS associates with transcriptional differences in BCAA transporters or catabolic genes in BAT. Neither transporter nor catabolic enzyme transcripts showed significant associations with LPS (Supplementary Figure S9), suggesting that higher BAT BCAA abundance associated with LPS was not explained by altered expression of these genes. These findings raise the possibility that post-transcriptional or mitochondrial mechanisms may contribute to the LPS–BCAA relationship.

Given that circulating LPS can influence mitochondrial function through Toll-like receptor 4 (TLR4) signalling [26], we next examined the relationship between circulating LPS and key components of its sensing pathway [27] in BAT. Among these, *LY96*, the co-receptor that binds LPS and enables TLR4 activation, showed a significant positive correlation with circulating LPS levels. Higher *LY96* expression was further associated with higher z-scores for the succinate-to-fumarate step of the TCA cycle (Figure 3D). Closer inspection revealed that *LY96* expression was specifically related to *SDHD* and the assembly factor *SDHAF2*, components involved in complex II stabilization [28], rather than other SDH subunits. Because these components are critical for SDH assembly and stability [28,29], this pattern may reflect complex II related transcriptional adaptation in response to mitochondrial stress, rather than enhanced enzymatic flux. Furthermore, the LPS adaptor *TRAF6* showed inverse correlations with z-scores of the α-ketoglutarate–to–succinyl-CoA and succinyl-CoA–to–succinate steps, whereas *NFKB1*, *NFKB2*, and *RELB* correlated positively with BCAA transporter expression (Figure 3D), suggesting a relationship between NF-κB signalling and transcriptional regulation of BCAA transport.

Together, these findings link metabolic endotoxemia to higher BAT BCAA and aminomalonate abundance and transcriptional changes involving BCAA and TCA-cycle metabolism. Such interplay between inflammatory and metabolic signalling may contribute to altered BCAA handling and mitochondrial function in BAT, with potential consequences for systemic BCAA homeostasis and insulin sensitivity in chronic metabolic inflammation.

### 3.6 Circulating BCAA trajectories over five years differ by BAT metabolism

**Figure 4:**
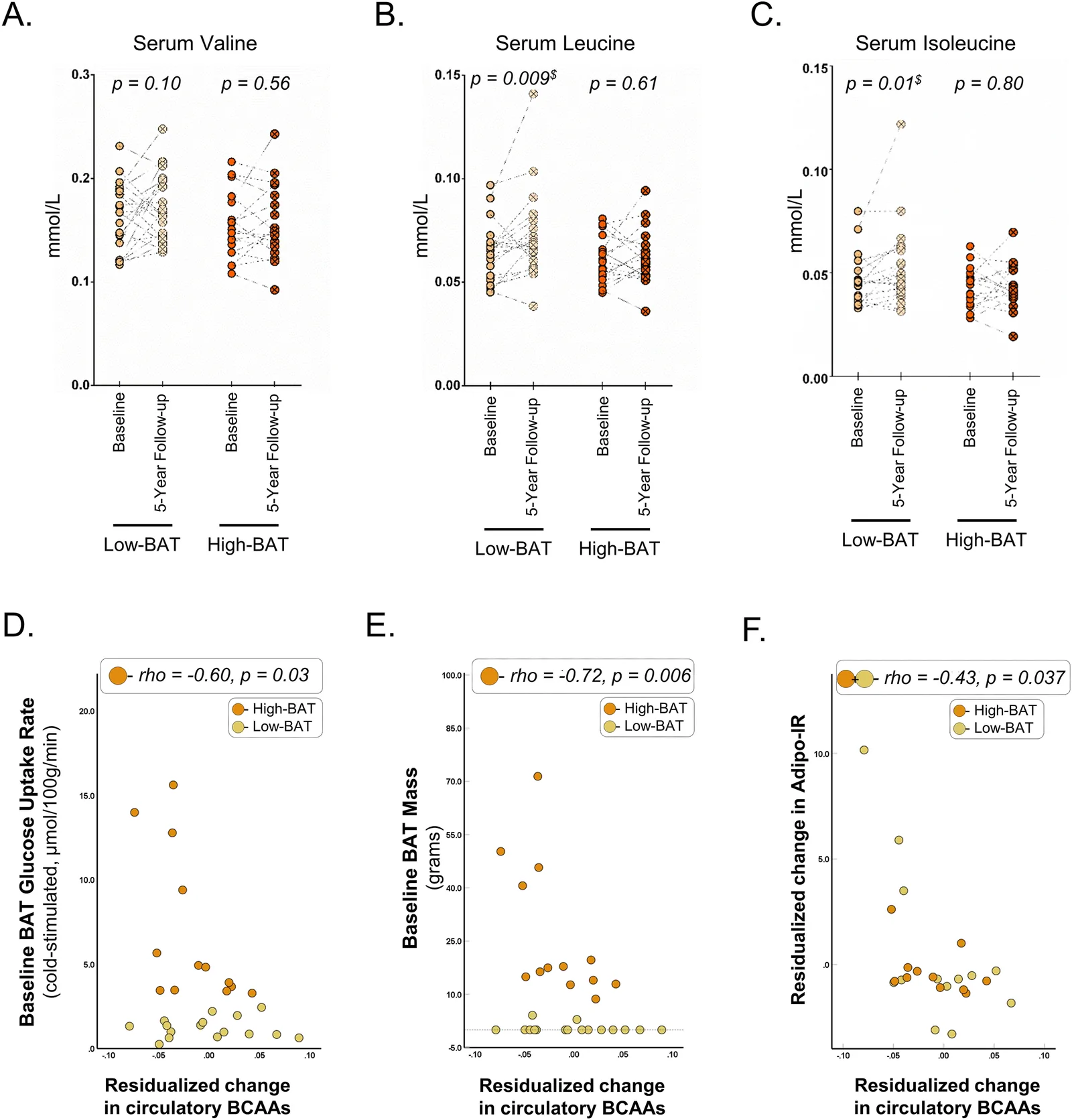
**Longitudinal changes in circulating BCAAs and their relationship with BAT and adipose tissue insulin resistance. A–C**. Changes in serum valine (A), leucine (B), and isoleucine (C) concentrations at baseline and after 5 years in individuals with High-BAT and Low-BAT metabolism. **D.** Correlation between baseline cold-stimulated BAT glucose uptake rate and residualized 5-year changes in circulating BCAA levels. **E.** Correlation between baseline BAT mass and residualized 5-year changes in circulating BCAA levels. **F.** Correlation between residualized 5-year changes in Adipo-IR and residualized changes in circulating BCAA levels. ^$^ Wilcoxon signed-rank test.

To assess the long-term relevance of BAT for systemic BCAA homeostasis, we re-contacted a subset of participants (*n* = 40, Supplemental Table S4) approximately five years after their baseline visit. Individuals with high BAT metabolism at baseline largely maintained stable circulating BCAA concentrations, whereas those with low BAT metabolism exhibited pronounced increases in leucine and isoleucine over the follow-up period (Figure 4A–C).

We next examined whether the observed longitudinal BCAA pattern was also evident across other circulating amino acids. Alanine and histidine increased over the five-year follow-up in both High-BAT and Low-BAT groups, whereas glutamine, glycine and phenylalanine did not change significantly in either group (Supplementary Figure S10). Thus, the BAT-group-related longitudinal pattern observed for BCAAs was not broadly reproduced across the other measured circulating amino acids.

Although circulating BCAA concentrations remained relatively stable at the group level in High-BAT participants, individual responses varied. We therefore examined whether baseline BAT characteristics were associated with longitudinal BCAA dynamics. To account for baseline differences and avoid changes being driven by extreme starting values (regression to the mean), we computed residualized change scores by regressing follow-up BCAA levels on baseline values. These residuals indicate whether an individual’s follow-up BCAA concentration was higher or lower than expected based on their baseline concentration. Within the High-BAT subgroup, both baseline cold-stimulated BAT glucose uptake and BAT mass inversely correlated with residualized BCAA values (BAT glucose uptake: *rho = −0.60, p = 0.03*; BAT mass: *rho = −0.72, p = 0.006*, Figure 4D–E). No corresponding associations were observed in low-BAT individuals, indicating that greater BAT metabolism is linked to smaller-than-expected increases in circulating BCAAs over time.

Across the entire cohort, residualized BCAA values also inversely correlated with residualized Adipo-IR (*rho = −0.43, p = 0.037*, Figure 4F). Thus, individuals whose BCAA levels increased more than predicted tended to show smaller-than-predicted increases in adipose tissue insulin resistance. Although this direction was unexpected given the established cross-sectional relationship between elevated BCAAs and insulin resistance, it suggests that longitudinal changes in circulating BCAAs and adipose tissue insulin resistance may not necessarily evolve in parallel.

Together, these findings show that greater BAT metabolic capacity is associated with more stable, or better-than-predicted, circulating BCAA trajectories over time, supporting a longitudinal relationship between BAT metabolism and systemic BCAA homeostasis.

## 4 Discussion

Our study links human BAT metabolic capacity with systemic and tissue BCAA homeostasis and identifies low-grade metabolic inflammation as a potential influence on this relationship. Individuals with high BAT metabolism exhibited lower circulating and BAT-resident BCAA levels, higher expression of BCAA catabolic genes, and relatively stable circulatory BCAAs concentrations over longitudinal follow-up. We further found that higher circulating LPS, reflecting metabolic endotoxemia, was associated with greater BAT BCAA and aminomalonate abundance, together with inflammatory and mitochondrial complex II-related transcriptional patterns. These findings support a role for BAT in human BCAA handling and suggest that low-grade inflammation may influence BAT-associated BCAA metabolism, with potential implications for nutrient metabolism, mitochondrial function, and systemic metabolic health.

**Figure 5:**
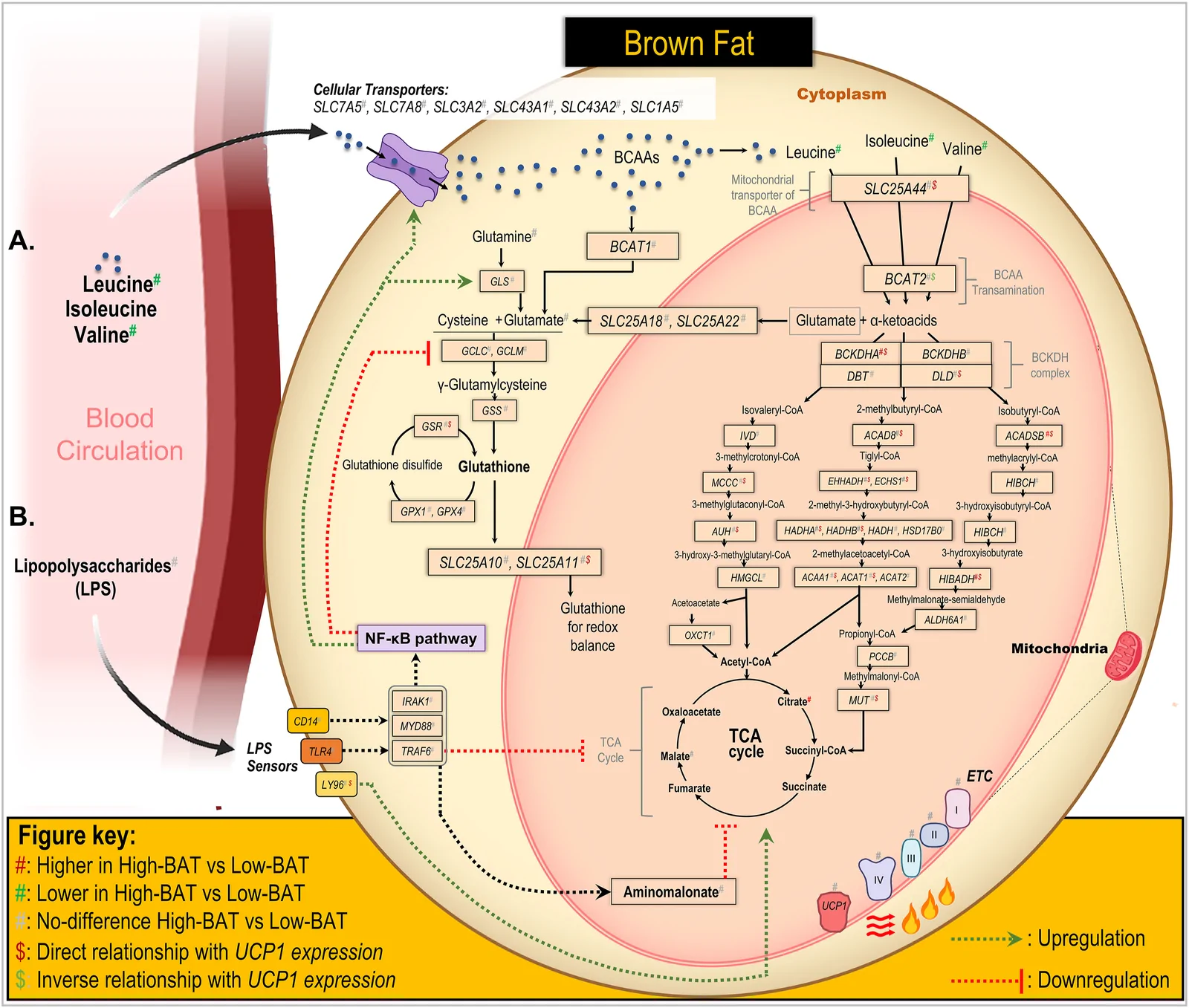
**Graphical summary of BAT BCAA metabolism and inflammatory signalling interactions**. **A.** Individuals with High-BAT metabolism show lower circulating and lower relative abundance of BAT-resident BCAAs, consistent with enhanced BCAA catabolism rather than differential uptake. Expression of key BCAA catabolic genes correlates with *UCP1*, suggesting that BCAA oxidation supports thermogenic activity. **B.** Proposed mechanism: Circulating lipopolysaccharides (LPS) activates LPS-sensing pathways in BAT, potentially stimulating NF-κB signalling. This pathway may upregulate BCAA transporters but perturb TCA cycle metabolism, contributing to mitochondrial stress and compensatory remodelling of complex II (SDH). LPS-signalling/NF-κB activation may also increase aminomalonate in BAT, potentially altering succinate handling at the SDH step and constraining BCAA oxidation. Concurrently, increased glutamine-to-glutamate conversion and reduced glutathione synthesis may weaken redox balance and promote inflammatory mitochondrial dysfunction. Dashed arrows indicate proposed relationships requiring functional validation.

Preclinical studies have shown that activated BAT can oxidize BCAAs and thereby influence systemic amino acid turnover [6]. Our findings extend this concept to humans by demonstrating that BAT exhibits a transcriptional profile consistent with enhanced BCAA catabolism, independent of transporter differences, and tightly aligned with *UCP1* expression (Figure 5A). Moreover, the lower relative BCAA abundance in High-BAT individuals was not accompanied by a generalized reduction across the other measured amino acids and was not similarly observed in adjacent WAT, suggesting that this metabolic phenotype is enriched in BAT. The observation that High-BAT individuals maintain lower circulating BCAA levels and favourable insulin sensitivity suggests that BAT may contribute to systemic amino acid regulation beyond its role in thermogenesis. Although skeletal muscle and liver remain dominant sites of BCAA oxidation, our data imply that the oxidative capacity of BAT could make a measurable contribution to whole-body BCAA clearance. Quantitative estimates (see Supplemental Information) indicate that even modest BAT oxidative activity could theoretically contribute to the BCAA concentration differences observed between insulin-sensitive and insulin-resistant individuals, highlighting BAT’s potential to influence circulating amino acid pools and metabolic homeostasis.

A particularly noteable finding of our study is the association between metabolic endotoxemia and the BAT metabolic phenotype. Higher circulating LPS was associated with greater BAT BCAA and aminomalonate abundance, together with NF-κB signalling-related and mitochondrial complex II-related transcriptional patterns. Aminomalonate is a non-proteinogenic amino dicarboxylate whose origin in mammalian tissues remains poorly understood. Early studies identified aminomalonate in alkaline protein hydrolysates from bacteria and human atherosclerotic plaques, and proposed oxidative modification of amino-acid residues or errors in protein synthesis as possible origins of protein-bound aminomalonate [30,31]. These studies, however, do not establish the origin of the aminomalonate signal detected in human adipose tissue. Microbial enzymatic pathways capable of aminomalonate biosynthesis have recently been identified [32], although their relevance to human tissue aminomalonate remains unknown. In the present study, this tissue signal could reflect endogenous metabolism, protein modification or turnover, microbial contributions, or exogenous exposure, none of which can be distinguished from the current data. Its structural similarity to malonate, together with its relationship with the succinate:fumarate ratio, prompted us to consider a possible connection with the SDH step. However, direct inhibition of SDH by aminomalonate has not been demonstrated, and our relative signal-intensity data cannot establish enzyme inhibition or whether tissue levels are functionally relevant. Taken together, these findings raise the possibility that metabolic endotoxemia contributes to a BAT state characterized by altered BCAA handling and mitochondrial metabolism, with potential consequences for BAT metabolic function. This proposed interaction is summarized in Figure 5B; however it requires direct functional validation.

Recent evidence suggests a bidirectional relationship between BCAA metabolism and lipid remodelling, with phosphodiesterase 3B (PDE3B) emerging as a molecular mediator linking BCAA-derived signalling to adipocyte lipolysis [33]. Consistent with this concept, we observed that BAT BCAA content was positively associated with local lipid accumulation measured by MR spectroscopy (*rho = 0.71, p = 0.02*). Furthermore, BAT BCAA levels correlated with eicosadienoic acid (C20:2 n-6), a product of omega-6 fatty acid elongation and desaturation, suggesting that altered PDE3B-dependent lipid mobilization may accompany changes in amino acid metabolism in BAT. Given PDE3B’s role in insulin-mediated suppression of lipolysis [34], disruption of this pathway could contribute to the co-accumulation of BCAAs and unsaturated fatty acids observed in metabolically compromised BAT.

BAT may modulate systemic BCAA homeostasis through multiple mechanisms. In addition to direct oxidative catabolism, recent isotope-tracing studies indicate that BCAA-derived nitrogen can contribute to the synthesis of non-essential amino acids and glutathione in brown adipocytes [10]. Consistent with this broader metabolic role, BAT-resident alanine abundance in our cohort was positively associated with the Matsuda index and inversely associated with muscle and adipose tissue insulin resistance, although these exploratory associations do not establish that the measured alanine was directly derived from BCAA catabolism. BAT can also release BCAA-derived metabolites, such as 3-hydroxyisobutyrate, as well as endocrine factors like fibroblast growth factor 21 (FGF21) [35,36], which in rodents regulate muscle metabolism and contribute to amino acid homeostasis [37]. Thus, endocrine and metabolite-mediated crosstalk between BAT and other metabolic tissues could further contribute to systemic BCAA regulation. Our five-year follow-up data support a longitudinal relationship: individuals with High-BAT metabolism maintained relatively stable circulating BCAA concentrations, whereas those with low BAT metabolism exhibited increases in leucine and isoleucine. Importantly, this pattern was not broadly reproduced across the other measured circulating amino acids, some of which changed similarly over time in both BAT groups. Given that elevated plasma BCAAs prospectively predict insulin resistance and type 2 diabetes in large-scale cohorts [38,39], therapeutic strategies to enhance BAT metabolism or reduce metabolic endotoxemia, such as cold exposure, pharmacologic BAT activators, or microbiome-modulating interventions, warrant further investigation as potential approaches to improve amino acid homeostasis and metabolic health.

Taken together, our results support an integrative framework connecting the gut, BAT, and systemic metabolism. Increased intestinal permeability or microbiome-derived LPS may be linked to inflammatory signalling in BAT, altered mitochondrial metabolism, and impaired BCAA handling, potentially reducing thermogenic flexibility and contributing to systemic metabolic dysfunction. This proposed gut-BAT-metabolism axis expands current paradigms centred predominantly on muscle and liver and provides a framework for investigating BAT as a contributor to systemic BCAA homeostasis.

## 5 Limitations and future directions

Our study is observational and cannot establish causality. Although we integrated imaging, metabolomics, and transcriptomic analyses, interventional studies are required to determine whether BAT activation or reduction of metabolic endotoxemia causally improves BCAA turnover and insulin sensitivity. Quantifying BAT’s absolute contribution to systemic BCAA oxidation relative to muscle or liver remains challenging.

A further limitation is that paired room-temperature-to-cold changes in circulating BCAAs could not be quantified because serum samples from the two conditions were analysed using different metabolomics platforms. Moreover, circulating amino-acid concentrations reflect whole-body amino-acid balance driven by multiple tissues and processes, and therefore cannot directly clarify BAT-specific BCAA disposal. Thus, even paired serum cold-response measurements would provide information on the systemic BCAA response rather than directly establish BAT-specific BCAA handling. A more direct assessment of acute-cold induced BAT BCAA handling would require dedicated tracer-based flux measurements (for example with dynamic PET acquisitions using BCAA-analogue radiotracers) and/or paired tissue profiling before and during cold stimulation. These approaches were not included in the present protocol, and repeated supraclavicular BAT biopsies during acute cold exposure remain ethically and logistically challenging in healthy volunteers.

The aminomalonate findings also warrant cautious interpretation. Aminomalonate was measured as a relative metabolomic signal rather than an absolute tissue concentration, and its biological source and metabolic fate could not be established. Furthermore, we did not measure SDH enzymatic activity, mitochondrial respiration, or TCA-cycle flux and therefore cannot determine whether aminomalonate influences succinate oxidation or whether the succinate:fumarate ratio reflects altered SDH activity.

Future studies should address dietary factors, microbiome composition, inter-organ signalling pathways, and directly quantify BAT BCAA flux and metabolite release. Nonetheless, the multi-layered evidence presented here provides a coherent framework for testing the translational relevance of the proposed gut–BAT–BCAA relationship.

## 6 Conclusions

In humans, BAT metabolic capacity is associated with systemic BCAA homeostasis, a stronger tissue BCAA-catabolic phenotype, and more favourable long-term circulating BCAA trajectories. Low-grade metabolic endotoxemia is additionally associated with altered BAT BCAA and aminomalonate abundance and mitochondrial-related transcriptional changes. These findings support a role for human BAT beyond thermogenesis as a potential contributor to systemic BCAA homeostasis and highlight links between inflammation, mitochondrial metabolism, and metabolic health.

## CRediT authorship contribution statement

**Mueez U-Din:** Writing – review & editing, Writing – original draft, Project administration, Methodology, Investigation, Formal analysis, Data curation, Conceptualization. **Francisco M. Acosta:** Writing – review & editing, Data curation. **Teemu Saari:** Writing – review & editing, Data curation. **Vanessa D. de Mello Laaksonen:** Writing – review & editing, Data curation. **Birgitta W. van der Kolk:** Writing – review & editing. **Milena Monfort-Pires:** Writing – review & editing. **Mercedes Clemente-Postigo:** Writing – review & editing, Investigation. **Tarja Niemi:** Writing – review & editing, Investigation. **Markku Taittonen:** Writing – review & editing, Investigation. **Marjo Tuomainen:** Writing – review & editing, Investigation. **Tobias Fromme:** Writing – review & editing, Data curation. **Marko Lehtonen:** Writing – review & editing, Data curation. **Karsten Kristiansen:** Writing – review & editing, Data curation. **John W. Newman:** Writing – review & editing, Investigation. **Kirsi H. Pietiläinen:** Writing – review & editing. **Jussi Pihlajamäki:** Writing – review & editing. **Eija Pirinen:** Writing – review & editing. **Martin Klingenspor:** Writing – review & editing, Investigation. **Kati Hanhineva:** Writing – review & editing, Investigation. **Juho Raiko:** Writing – review & editing, Project administration, Methodology, Investigation, Conceptualization. **Kirsi A. Virtanen:** Writing – review & editing, Supervision, Resources, Project administration, Methodology, Investigation, Funding acquisition, Conceptualization.

## Ethics declaration

Written informed consent to take part in the study and to publish the article has been obtained from all participants or their legal representatives. The privacy rights of participants have been observed.

This study included organ or tissue donors. This study includes human biological material and consent was obtained by donors, or their next of kin or legal representatives, for use in this study and for publication of the article. The samples used in this research were not sourced from executed prisoners or prisoners of conscience.

This study was performed in compliance with relevant laws, regulatory frameworks and guidelines where the research took place. This study was approved by the Ethical Committee of South-West Finland Hospital District. (Approval No. T4/2011 Nro 14824)

## Declaration of generative AI and AI-assisted technologies

During the preparation of this manuscript, the authors used ChatGPT (version 5.2 & 5.4), Grammarly and Grok in order to improve the readability of the text. The animated study participants shown in the graphical abstract have been produced with Leonardo AI image generation tool; however, rest of the graphical abstract have been designed by the authors. After using these tool or services, the authors reviewed and edited the content as needed and take full responsibility for the content of the publication.

## Declaration of competing interest

The authors declare no competing interests.

## Data Availability

Data will be made available on request.
