# Supplementary material for: Human brown fat metabolism associates with systemic branched-chain amino acids homeostasis
Source: Mol Metab. 2026 Aug 26;112:102434. doi: 10.1016/j.molmet.2026.102434 (PMC13571953; doi:10.1016/j.molmet.2026.102434)
Supplement: Supplementary file 1 [file mmc1.docx]

***Supplemental Information***

**Human Brown Fat Metabolism Associates with Systemic BCAA Homeostasis**

**Authors:**

Mueez U-Din¹, Francisco M. Acosta¹^,2,3^, Teemu Saari¹, Vanessa D. de Mello Laaksonen^4^, Birgitta W van der Kolk^5^, Milena Monfort-Pires¹, Mercedes Clemente-Postigo^6,7,8^, Tarja Niemi^9^, Markku Taittonen^10^, Marjo Tuomainen^4^, Tobias Fromme^11^, Marko Lehtonen^12^, Karsten Kristiansen^13^, John W. Newman^14^, Kirsi H. Pietiläinen^3^, Jussi Pihlajamäki^4,15^, Eija Pirinen^16^, Martin Klingenspor^11^, Kati Hanhineva^17^, Juho Raiko¹, Kirsi A. Virtanen¹*

| **Table S1.** Main characteristics of the participants (entire study cohort) | | | |
| --- | --- | --- | --- |
|  | All subjects | High-BAT | Low-BAT |
| N | 83 | 46 | 37 |
| Age, years | 39.2 ± 9.8 | 35.8 ± 10.3 | 42.8 ± 8.1^#^ |
| Male / female, n | 24 / 58 | 8 / 38 | 16 / 21 |
| BMI, kg/m^2^ | 26.8 ± 5.5 | 24.9 ± 3.8 | 29.3 ± 6.3^#^ |
| Whole-body fat percentage, % | 32.4 ± 9.4 | 31.6 ± 7.9 | 33.5 ± 11.0 |
| Waist, cm | 89.4 ± 16.9 | 83.7 ± 12.9 | 96.2 ± 18.6^#^ |
| Insulin sensitivity (M-value) (μmol/kg/min) | 40.4 ± 20.8 | 46.8 ± 19.8 | 31.7 ± 19.3^#^ |
| Data are mean ± SD  ^#^ Statistically different from High-BAT group based on Independent Sample Student t-test or Mann-Whitney U test | | | |

| **Table S2.** Main characteristics of the participants providing BAT biopsies for **metabolomics** | | | |
| --- | --- | --- | --- |
|  | All subjects | High-BAT | Low-BAT |
| N | 17 | 10 | 7 |
| Age, years | 39.0 ± 9.5 | 36.5 ± 9.4 | 42.6 ± 9.3 |
| Male / female, n | 4 / 13 | 2 / 8 | 2 / 5 |
| BMI, kg/m^2^ | 25.6 ± 3.6 | 24.2 ± 2.5 | 27.5 ± 4.2 |
| Whole-body fat percentage, % | 32.3 ± 8.2 | 31.7 ± 6.5 | 33.2 ± 10.6 |
| Waist, cm | 84.4 ± 14.5 | 79.6 ± 11.0 | 91.3 ± 16.8 |
| Insulin sensitivity (M-value) (μmol/kg/min) | 36.8 ± 21.4 | 39.0 ± 18.0 | 33.2 ± 27.6 |
| Data are mean ± SD | | | |

| **Table S3.** Main characteristics of the participants providing BAT biopsies for **RNA-sequencing** | | | |
| --- | --- | --- | --- |
|  | All subjects | High-BAT | Low-BAT |
| N | 14 | 9 | 5 |
| Age, years | 38.3 ± 10.2 | 36.7 ± 10.2 | 41.2 ± 10.6 |
| Male / female, n | 3 / 11 | 2 / 7 | 1 / 4 |
| BMI, kg/m^2^ | 25.7 ± 4.7 | 23.8 ± 2.6 | 29.2 ± 5.8 |
| Whole-body fat percentage, % | 31.7 ± 9.8 | 29.9 ± 7.8 | 35.0 ± 13.0 |
| Waist, cm | 84.4 ± 17.1 | 76.8 ± 9.7 | 98.0 ± 20.0 |
| Insulin sensitivity (M-value) (μmol/kg/min) | 40.7 ± 28.2 | 52.1 ± 27.3 | 17.8 ± 11.2 |
| Data are mean ± SD | | | |

| **Table S4.** Main characteristics of the participants in **Follow-up group** | | | | | | |
| --- | --- | --- | --- | --- | --- | --- |
|  | **Baseline** | | | **Follow-up (5 – years)** | | |
|  | All subjects | High-BAT | Low-BAT | All subjects | High-BAT | Low-BAT |
| N | 40 | 18 | 22 | 40 | 18 | 22 |
| Age, years | 41.3 ± 7.4 | 38.9 ± 8.0 | 43.2 ± 6.5 | 46.7 ± 7.2* | 45.1 ± 7.7* | 47.9 ± 6.7* |
| Male / female, n | 11 / 29 | 3 / 15 | 8 / 14 | 11 / 29 | 3 / 15 | 8 / 14 |
| BMI, kg/m^2^ | 28.0 ± 6.4 | 25.0 ± 4.1 | 30.4 ± 6.9 | 28.3 ± 6.5* | 26.0 ± 4.7 | 30.1 ± 7.2 |
| Whole-body fat perct. % | 34.2 ± 9.8 | 32.3 ± 8.2 | 35.7 ± 10.8 | 35.2 ± 9.6* | 34.8 ± 7.3* | 35.6 ± 11.2 |
| Waist, cm | 91.1 ± 18.6 | 82.9 ± 12.9 | 97.3 ± 20.1 | 97.7 ± 18.4* | 91.8 ± 14.3* | 101.8 ± 20.2* |
| Adipo-IR | 4.5 ± 4.5 | 3.3 ± 2.5 | 5.5 ± 5.7 | 5.6 ± 5.6* | 3.5 ± 2.3* | 7.3 ± 6.9* |
| HOMA-IR | 1.6 ± 1.3 | 1.3 ± 0.8 | 1.8 ± 1.6 | 2.1 ± 1.6* | 2.0 ± 1.5* | 2.2 ± 1.7* |
| Data are mean ± SD  Follow-up measurements: AdipoIR (n = 24), HOMA-IR (n = 22)  * Statistically different than the corresponding paired-group based on Paired Sample Student t-test or Wilcoxon-rank sum test | | | | | | |

**Supplementary Figure S1. Serum amino acids, other than BCAAs, according to BAT metabolic status.**

Serum concentrations of available amino acids, other than BCAAs, measured at room temperature (RT) are shown for individuals with Low-BAT and High-BAT metabolism. Alanine, glutamine, glycine, histidine and phenylalanine were quantified using the Nightingale Health ^1^H-NMR platform and are presented in mmol/L. Glutamate measured during acute cold exposure was measured with UHPLC–qTOF-MS and is presented as signal intensity. Box plots show median and interquartile range; whiskers indicate minimum–maximum values. Individual data points are shown.

**Supplementary Figure S2. BAT-resident non-BCAA amino acids according to BAT metabolic status.**

Signal intensities of measured non-BCAA amino acids in supraclavicular brown adipose tissue (BAT) are shown for individuals with Low-BAT and High-BAT metabolism. Amino acids were measured by tissue metabolomics and are presented as signal intensity. Box plots show median and interquartile range; whiskers indicate minimum–maximum values. Individual data points are shown.

**Supplementary Figure S3. WAT-resident BCAA abundance according to BAT metabolic status.**

(A) Signal intensities of valine, leucine and isoleucine in white adipose tissue (WAT) from individuals with Low-BAT and High-BAT metabolism. (B) Relative signal intensities of WAT-resident BCAAs, calculated as the signal intensity of each BCAA divided by the summed signal intensity of all measured amino acids in WAT. Box plots show median and interquartile range; whiskers indicate minimum–maximum values. Individual data points are shown.

**Supplementary Figure S4. WAT-resident amino acids, other than BCAAs, according to BAT metabolic status.**

Signal intensities of measured amino acids, other than BCAAs, in white adipose tissue (WAT) are shown for individuals with Low-BAT and High-BAT metabolism. Box plots show median and interquartile range; whiskers indicate minimum–maximum values. Individual data points are shown.

**Supplementary Figure S5. WAT expression of BCAA transport and catabolic genes according to BAT metabolic status.**

Log_2_ fold differences in the expression of genes involved in BCAA transport, transamination, BCKDH complex activity, and downstream catabolic pathways, are shown in white adipose tissue (WAT) from High-BAT versus Low-BAT individuals. The right-side column shows Spearman correlations between WAT gene expression and WAT *UCP1* expression. Dots represent log_2_ fold difference between High-BAT and Low-BAT groups, and error bars indicate SEM. Genes with significant differences are denoted with * (p < 0.05) and trend-level p values are shown for p < 0.10. None of the genes showed a significant relationship with *UCP1* expression in WAT.

**Supplementary Figure S6. Correlations between BAT-Resident BCAAs, BCAA Metabolism Genes, and Systemic Metabolic Indices**

**A.** Correlations BCAAs in BAT and indices of whole-body insulin sensitivity, insulin resistance, and glucose homeostasis. **B.** Correlations between BAT genes involved in BCAA metabolism and indices of whole-body insulin sensitivity, insulin resistance, and glucose homeostasis. Significant associations are indicated by yellow markers (• p < 0.05, ◆ p < 0.01).

**Supplementary Figure S7. Associations of BAT-resident amino acids known to synthsize from BCAA-derived nitrogen with insulin sensitivity measures.**

Spearman correlations of BAT-resident glutamate, alanine, proline and glycine signal intensities with whole-body insulin sensitivity assessed by the M-value and Matsuda index, and with HOMA-IR, adipose tissue insulin resistance (Adipo-IR), muscle insulin resistance (MIRI) and hepatic insulin resistance (HIRI). Yellow markers indicate significant associations (p < 0.05).

**Supplementary Figure S8. Relationship between circulating LPS and WAT-resident BCAA abundance.** Spearman correlations between circulating lipopolysaccharide (LPS) concentrations and (A) summed BCAA signal intensity, (B) valine, (C) leucine, and (D) isoleucine signal intensities in white adipose tissue (WAT). No significant associations were observed between circulating LPS and WAT-resident total or individual BCAAs.

**Supplementary Figure S9. Correlations between circulatory lipopolysaccharide (LPS) and BAT genes involved in BCAA metabolism.** Significant associations, if any, are indicated by yellow markers (• p < 0.05, ◆ p < 0.01, ✸ p < 0.001).

**Supplementary Figure S10. Five-year changes in circulating amino acids other than BCAAs according to BAT metabolic status.**

Serum concentrations of alanine, glutamine, glycine, histidine and phenylalanine at baseline and approximately five-year follow-up are shown separately for individuals with Low-BAT and High-BAT metabolism. Individual participants are connected by dotted lines. Alanine and histidine increased over follow-up in both BAT groups, whereas glutamine, glycine and phenylalanine showed no significant within-group changes. P values indicate paired comparisons between baseline and follow-up within each BAT group.

**Illustrative calculation of BAT contribution to circulating BCAA clearance**

Using a serum volume of ~2.6 L, the difference in circulating BCAAs between a metabolically healthy and an insulin-resistant exemplar in our cohort (Δ valine 0.24 mmol·L^-1^, Δ leucine 0.06 mmol·L^-1^, Δ isoleucine 0.08 mmol·L^-1^) corresponds to a total extra pool of ≈ 0.99 mmol or ≈ 0.12 g of BCAAs in blood. A 250-g depot of activated BAT with an O_2_ consumption of 0.6 – 4.0 mL.100g^-1^.min^-1^ (U Din et al., 2018) would consume O_2_ ≈ 1.5 – 10 mL.100g^-1^.min^-1^ (≈ 6.7×10^-5^ to 4.5×10^-4^ mol O_2_·min^-1^). If that oxidative flux were directed to complete BCAA oxidation (stoichiometric O_2_ requirements ≈ 6.8 mol O_2_·mol^-1^ for valine and ≈ 8.3 mol O₂·mol^-1^ for leucine/isoleucine), the implied BCAA oxidation capacity of BAT would be on the order of **~3 – 22 mg total BCAA·min**^-1^ across the low-to-high O_2_ range. At these rates, the calculated excess circulating pool (~0.12 g) could be cleared in roughly **~5 – 40 minutes** (best- to worst-case, upper-bound estimate).

These back-of-the-envelope calculations show that, under idealized assumptions, even modest BAT oxidative activity is plausibly sufficient to produce measurable changes in circulating BCAAs on a biologically relevant timescale. Crucially, the estimates assume complete oxidation of BCAAs, exclusive allocation of BAT oxidative flux to amino-acid oxidation (no competition from fatty acids or glucose), and a static serum pool; in vivo fluxes will be modulated by interorgan exchange, diet, renal clearance and non-oxidative BCAA fates. Thus the arithmetic supports plausibility but not quantitative proof that BAT alone accounts for steady-state BCAA differences, tracer-based flux measurements are required for definitive partitioning.
